# Are dietary intake and nutritional status influenced by gender? The pattern of dietary intake in Lao PDR: a developing country

**DOI:** 10.1186/s12937-020-00545-9

**Published:** 2020-04-11

**Authors:** Kethmany Ratsavong, Tessa van Elsacker, Daovieng Doungvichit, Latsamy Siengsounthone, Sengchanh Kounnavong, Dirk Essink

**Affiliations:** 1 Lao Tropical and Public Health Institute (Lao TPHI), Vientiane, Laos; 2 Athena Institute, Vrije University, Amsterdam, the Netherlands; 3 Ministry of Health, Vientiane, Laos

**Keywords:** Gender, Dietary intake, Dietary adequacy, Women, Children, Lao PDR, 24-h recall

## Abstract

**Background:**

Recognition of discrepancies between men and women in nutritional intake is important to tackle food and nutrition insecurity and the often-double burden of malnutrition. The purpose of this study was to assess nutritional status and dietary intake of the Lao population, with a focus on possible influences of gender.

**Methods:**

Dietary intake was assessed in a national cross-sectional study of 1771 randomized participants aged from 1.01 to 89 years, using 24-h dietary recall. Dietary reference intakes were used to assess nutrient insufficiency. Chi-square test was used to evaluate gender differences and multiple univariate logistic regression to examine associations between gender, nutritional status, demographics and nutrient insufficiency.

**Results:**

Nutrient insufficiencies were higher among pregnant and lactating women than other adult men and women, especially for protein and micronutrients such as vitamin B3, B1, C and other vitamins. Dietary intake and BMI were similar between men and women; all had insufficient intake of all types of nutrients, except sodium. However, women had lower intake than men for almost all nutrients and age groups. The prevalence of overnutrition was higher among those aged 18 years and over for both sexes. Among adult women (15–49.9 years old) and older adult women (50 years old or above), the proportions were: underweight 8.6% (both groups), overweight 18.4 and 20.5%, and obese 34.2 and 39.1%, respectively. Among pregnant and lactating women, the rates of underweight were 7.5 and 1.4%, of overweight were 17.8 and 27.1%, and obese, 21.9 and 40.0%. Among adult and older men, 3.2 and 8.3% were underweight; 21.0 and 18.6% were overweight and 28.2 and 27.6% were obese. Multiple univariate logistic regressions revealed that the factors rural area, dry season and Northern-Lowland region were associated with inadequate micronutrient intake among children, adolescents and adults of both genders.

**Conclusions:**

Dietary intakes were alarmingly micronutrient-insufficient. Macronutrient imbalance and double burden of malnutrition were confirmed in both sexes. Gender differences were limited; men and women had similarly insufficient intakes, but pregnant and lactating women were disproportionately affected. Nutritional interventions should also take men and older people into account to solve nutrition problems.

## Background

In low- and middle-income countries (LMICs), women are often found to be disproportionately affected by food and nutrition insecurity (FNI) and the ‘double burden of malnutrition’ (DBM), [1]. The global prevalence of FNI is on the rise. In 2017, about 10% of the world population was severely food insecure, compared to 8.4% in 2015 [2]. FNI, and related dietary inadequacy, may lead to various forms of malnutrition, ranging from undernutrition, such as underweight, stunting and wasting, to overweight and obesity [3]. Many LMICs now face the ‘double burden of malnutrition’ characterized by the coexistence of undernutrition, including micronutrient deficiencies, and overweight/obesity [4],which especially concerns women and girls.

Research reports suggest that women in households vulnerable to FNI are at greater risk of malnutrition than men in the same households [5]. Limited information is available on underweight in LMICs, however the latest WHO estimates indicate that the prevalence of underweight was slightly higher among women (17.1%) than men (16.6%) in 2017 [6].

Moreover, many diets in LMICs have been changing significantly in recent years, resulting in excessive weight gain in both men and women, with greater impact among women [7]. Prevalence of overweight and obesity were respectively 5 and 8.5% among men and 9.5 and 12.5% among women; these gender disparities may be increasing [7].

Men and women have different dietary requirements, which can be defined by sex and gender [8]. For instance, in Southeast Asia, the recommended dietary allowance (RDA) for protein is higher for men (48 g/day) than for women (40 g/day), while the RDA for iron is much higher for women of reproductive age (39 mg/day) compared to men (18 mg/day) [9]. Social and economic inequalities between men and women can negatively influence nutritional intake of Southeast Asian women, when men and boys are given priority to eat more nutritious food [10]. Also, as primary caregivers and food providers, women often feed their children before themselves [11] .

Previous research on gender inequality in reproductive age found that inadequate intakes of micronutrients like vitamin A and iron were more common among women than men [12]. However, in LMICs, where food is not available for everyone and men have three to four times the body size, exercise level, basal metabolic rate (BMR) and thus higher nutrient requirements than women [13, 14], it is possible that men are also malnourished. Recognition and understanding of discrepancies between men and women in nutritional intake will provide a more detailed understanding in how to tackle FNI and DBM in Laos.

As in other LMIC, in Lao PDR women are found to be disproportionately affected by nutrition problems. To improve the nutritional status of the population, the Ministry of Health developed a National Nutrition Strategy to 2025, which focuses mostly on children under 5, women of reproductive age, and pregnant and lactating women [15]. A 2013 national cross-sectional study on Lao adults men and women showed that prevalence of underweight, overweight and obesity were higher among women (10.4, 18.1 and 28.0%) than men (8.6, 16.6, and 21.4%) [16]. Pregnant and lactating women are particularly vulnerable to inadequate dietary intake due to cultural practices around pregnancy and lactation in Lao PDR, such as eating less and avoiding certain foods [17]. The National Food Consumption Survey, Lao PDR (2016), found that many people had significantly lower energy intakes than recommended [18]. This included lower dietary intakes among pregnant and lactating women, while they have certain dietary requirements that are significantly higher compared to men and non-pregnant/non-lactating women [19]. Additionally, in Lao PDR, differences in decision-making authority related to gender within families can increase vulnerability to FNI among Lao women, since men are usually allowed to make the final decision and, in some ethnic groups, men and boys are allowed to eat before women and girls [20]. Thus, gender disparities are hypothesized to play a major role in potential differences in dietary intakes between Lao men and women. However, the national estimates did not explore gender differences in dietary intake as an outcome, which makes it unclear whether and to what extent dietary intakes of Lao men and women differ.

Despite the concern about sex and gender discrepancies in the prevalence of FNI and the DBM in Lao PDR, the differences in dietary intake between Lao men and women remains unexplored. The information on dietary intake and its influence on the nutritional status of both sexes might explain the disparity. Therefore, this study aimed to determine whether gender influenced dietary intake and its associations with BMI and related variables in the population of Lao PDR. We also looked at differences among different age groups, since each life stage has specific dietary requirements**.** It was hypothesized that Lao women and girls of all age groups are less able to meet their dietary recommendations than men and boys. Especially, dietary intakes of women of reproductive age (including pregnant and lactating women) are expected to differ from those of adult men. A better understanding of dietary intakes and possible gender differences among different age groups of the Lao PDR population could be used to optimize current and design new nutritional interventions aim at improving dietary intakes.

## Methods

### Study design and setting

This study was carried out as a quantitative cross-sectional exploratory study, a national food consumption survey (FCS). All participants were recruited from two representative provinces in each of the three regions of the country with common features of geography and agriculture: Northern Lowland (Oudomxay, *n* = 295 and Luangprabang, *n* = 303), Central-Southern Highland (Vientiane city, *n* = 301 and Bolikhamxay, *n* = 287), and Mekong Corridor (Savannakhet, *n* = 294 and Champasack, *n* = 291). Multistage stratified cluster sampling was used to select a randomly represented study sample in each region; two districts in each province, two villages in each district and randomly selected households in each village. Two rounds of data collection were carried out to capture seasonal variation, since food intake is generally higher in the rainy season; the first round ran from 25 September to 15 December 2016 (rainy season) and the second from 13 March to 20 May 2017 (dry season) [21]. The same sampling methods were used for both rounds, leading to selection of different households in each round. In total, 2045 participants were included, 1021 in the rainy season and 1024 in the dry season, Table 1.

**Table 1:** Sampling method and numbers of participants per village/district/province in the FCS

Province	District	Round 1 Rainy season (Sept to Dec, 2016)			Round2 Dry season (March to May, 2017)		
		Village	Day 1 (*n*)	Day 2 (*n*)	Village	Day 1 (*n*)	Day 2 (*n*)
**Vientiane Ca**^a^	Hatxaiphone	Thapha	45	18	Dongphonehea	43	18
		Hatxaikhao	41		Nong Heo	42	
	Pakngeum	Phao	43		Natham	42	
		Maknaodong	41		Nafai	47	
**Oudomxay**	Hun	Langjing	42	18	Phonsavang	42	
		Nahom	43		Fan	45	
	Beng	Xienglear	34		Namat	42	19
		Samkang	53		Yor	43	
**Luangprabang**	Nambak	Makphout	45		Phonexieng	46	
		Pakmong	44		Phonexay	44	
	PakOu	Samsanouk	43	19	Hauylo	42	18
		Hatmat	42		Hatpang	42	
**Bolikhamxay**	Thabok	Palai	42		Saifai	42	21
		Nakham	42		Thauyai	42	
	Pakkading	Phonsy	42		Namkou	42	
		Sensamlan	42		Hatsaikham	42	
**Champasack**	Champasack	Nongthon	42		Nongsa	42	19
		Nasavang	42	17	Meuy	42	
	Paksong	Vatluang	42		Nonglea	42	
		Lak 40	42		Sepien	42	
**Savannakhet**	Atsaphangthong	Donepalai	42		Saphankeo	42	
		Huameuang	42	18	Pongdong	42	
	Songkhone	Lahakog	43		Nongokhiean	42	18
		Thakhamlien	42		Kongsaatt	42	

^a^Vientiane Capital

### Target population

The FCS study divided the target population into 4 different target groups: 1) children under six divided into 3 sub-groups, infants (3 to 11 months), toddlers (12 to 35 months), and pre-school children (3 to 5.9 years old); 2) adolescents; 3) all adult population 15 to 49.9 years old, with separate count for pregnant and lactating women as this group needs specific nutritional food; and 4) older adults, 50 years old and over. The sample for each age group was stratified by sex to represent females and males equally as shown in Table 2.

**Table 2:** Target population for the FCS study

Population group		Region			Total
		Mekong Corridor	Central-Southern highland	Northern Lowland	
**Infants: 3–11 months**	Male	48	48	48	144
	Female	48	48	48	144
**Toddlers: 12–36 months**	Male	48	48	48	144
	Female	48	48	48	144
**Children: 3–5.9 yrs.**	Male	48	48	48	144
	Female	48	48	48	144
**Adolescents: 6–14.9 yrs.**	Male	48	48	48	144
	Female	48	48	48	144
**Adults: 15–49.9 yrs.**	Male	48	48	48	144
	Female	48	48	48	144
**Older adults: ≥50 yrs.**	Male	48	48	48	144
	Female	48	48	48	144
**Pregnant women**	Female	48	48	48	144
**Lactating women**	Female	48	48	48	144
Sub-total		672	672	672	
Total		2016			

### Data collection materials

Demographic and anthropometric data were collected; to measure dietary intake, the World Health Organization (WHO) 24-h dietary recall (24hDR) questionnaire was used, adapted and translated into Lao language by the Lao Tropical and Public Health Institute (Additional file 1). Additionally, a tool set was used comprising a Food Photo Book, the Local Mixed Dishes Book, a Food Code Book and the Local Dish Database. The first two books were used during the interviews to help the interviewees to estimate food portion size (Food Photo Book) and to remember the consumed food ingredients of popular local menus (Local Mixed Dishes Book). The Food Code Book was used for coding food items of the 24hDR questionnaire, and the Local Dish Database to calculate the quantity of ingredients. In addition, weight and height of the participants were measured and collected by trained anthropometrist using a weighing scale (SECA 874 U) and children’s recumbent length (SECA 417) and adult height (SECA 213).

### Procedure

Before going to the field, we trained the data collector, who had experience in field data collection and a health and nutrition background, so we can expect the same standard for each enumerator. In each target village, adult individual subjects and/or mothers/primary caregivers (persons who are not the mother but have responsibility for feeding and care of the child) of the children were asked whether they were willing to participant in the study. Participants signed informed consent forms when they agreed to join the study and before interviews started. Face to face interviews using a standard set of questionnaires, including socio-demographic data and 24-h recall were used to record data. A trained anthropometrist also recorded the weight and height of all participants.

For the 24-h recall questionnaire, interviewees were asked to recall all items of food and drink consumed in the last 24 h, from waking up until going to bed the previous day. The questions also asked about an estimate of the type and amount of food, food patterns and the differences on weekends, special events or during sickness, to distinguish bias from general food and special feeding. None of the respondents reported being ill at the time of the survey.

The average portion size of each type of food was determined using commonly used household utensils and the Food Photo Book with contextualized local food pictures and weights of each portion. This facilitated estimation of food portion sizes and ingredients and enabled collection of the most precise estimates of the quantities of real food consumption of each food item, reducing errors in estimation of portion size. These results were analyzed together with the recorded set of anthropometric data.

### Subjects

For this study, participants younger than 1 year were excluded, as we focused on the influence of dietary intake related to nutritional status and gender. Most of the nutrient intake of children up to 1 year comes from breast milk and milk substitutes, and these very young children cannot choose food but depend on the primary caretaker, which would not fit with the research aim.

Finally, 1771 participants were included for data analysis. These participants were divided into seven life stage groups: 1) toddlers (1–2.9 years), 2) children (3–5.9 years), 3) adolescents (6–17.9 years), 4) adults (18–49.9 years), 5) older adults (> 50 years), 6) pregnant women, and 7) lactating women. All age groups, except pregnant and lactating women, included both men and women.

### Data processing and data analysis

The demographic and anthropometric data were recorded using Epidata software version 12. The food items from the 24hDR questionnaires were recoded according to the Food Code Book, entered and converted to nutrient intakes using ‘Inmucal- N3’ software copyright: 2007, Institute of Nutrition Mahidol University, Thailand which translates food items into nutrients using a Thai Food Composition Table [22]. Finally, all data were converted into SPSS data files for further analysis.

For both sexes and all age groups, demographic and anthropometric characteristics of the study population are presented as frequencies (%) for categorical variables (region, area of residence, season and nutritional status,) and as means and standard deviation (±SD) for continuous variables (age, weight and height). The mean and (±SD) were used to assess the nutrient adequacy when the distribution is a normal distribution, while medians interquartile range (IQR) were used when the distributions were not normal.

Since eight micronutrients were evaluated, the number of adequate micronutrient intakes ranged from 0 to 8, where ‘0’ indicated having no adequate intakes of micronutrients and ‘8’ having adequate intakes of all eight micronutrients. Micronutrient adequacy was defined as ‘adequate’ when four or more micronutrient intakes were adequate, and as ‘inadequate’ when four or less micronutrient intakes were adequate.

Proportions (%) of subjects with “insufficient nutrient intake” meaning all the nutrient intake lower than the range of ‘Dietary Reference Intakes’ (DRIs); “sufficient nutrient intake” meaning all the nutrient intake meet the DRIs range; and “excessive nutrient intake” meaning all nutrient intake above the DRIs range were calculated and compared between sexes. Chi-square tests were used to determine the significance of differences in nutrient insufficiencies between the sexes, as well as between pregnant/lactating women compared with adult men and with non-pregnant/non-lactating women (since DRIs of some nutrients differ between these life stage groups [23]. *P*-values < 0.05 were considered statistically significant.

To determine the association between sex and micronutrient inadequacy, univariate logistic regression analyses were conducted. For each age group, crude and stratified analyses for the area of residence (urban/rural) were performed. Results are expressed as ORs for inadequate micronutrient intake. For pregnant and lactating women, the ORs were presented as lactating-to-men/non-lactating women and pregnant-to-men/non-pregnant women ORs, respectively.

To evaluate the association of nutritional status and various demographic characteristics (area of residence, season and region) with insufficient and excessive nutrient intakes of Lao people, multiple univariate logistic regression analyses were performed. All statistical tests were performed for each age group separately, stratified by gender. *P*-values < 0.05 were considered statistically significant. All analyses were performed using SPSS version 24.0.

### Dependent variable: energy intake

The key outcomes in this analysis were differences in dietary intake, total energy intake and micro/macro-nutrient intake among men and women. Sufficiency of dietary intake was assessed by comparing the energy and macro- and micronutrient intakes of the participants to the recommendations of the guide ‘Dietary Reference Intakes’ (DRIs). The DRI covered the components: Estimated Energy Requirement (EER), the Acceptable Macronutrient Distribution Ranges (AMDRs), the Estimated Average Requirement (EAR) and Adequate Intake (AI), and, when available, the Tolerable Upper Intake Level (UL) [23]. Energy intake of the participants was compared to the Estimated Energy Requirement (EER) based on gender, age, weight, height and physical activity level (PAL), and therefore individually calculated for each participant by prediction equations [22]. Since most Lao people work in the agricultural sector [24] and an active PAL is recommended to maintain health [23], this PAL was used in the equations to estimate EERs, (Additional file 2).

### Independent variable: demographic and nutritional status

Body mass index (BMI): a measure of weight relative to height was calculated as weight (kg) for height (m), universally expressed in units of kg/m^2^. Using Asia-Pacific cut-off values, we categorized the results into four groups: BMI less than 18.5 kg/m^2^ was considered underweight, BMI between 18.5 and 22.9 kg/m^2^ as normal weight, BMI from 23 to 24.9 kg/m^2^ as overweight, and BMI more than ≥25 kg/m^2^ as obese [25]. For toddlers, children and adolescents, BMI was evaluated using WHO’s BMI-for-age Z-scores, categorized as BMI-Z-score less than <−2 being underweight, BMI-Z-score between − 2 and + 1 as normal weight, BMI-Z-score more than (> + 1) as overweight, and BMI-Z-score more than (> + 2) as obese [26].

### Results

Among 1771 participants, 1041 (58.8%) were women. In each age and gender group, the range of ages was almost the same. They represented the three regions and the majority lived in rural areas. About half of the dietary intakes were measured during the rainy season (Table 3).

More than 70% of the children, both boys and girls, had normal BMI. The only significant differences between the sexes were found in adolescent boys, with higher prevalence of underweight (9.3%) and obese (8.4%) than girls in the same age groups (*p* < 0.01). But starting from adults and older adults of both genders, the proportions of normal BMI decreased to less than 50% of the population. The key decrease was in malnutrition (both over-nutrition and undernutrition), which was higher in adult and older women compared to men in the same age groups. Both pregnant and lactating women also had a high proportion of over-nutrition, with more than 18% overweight and more than 20% obese (Table 3).

**Table 4:** Insufficient energy intake by sex per age group of active persons

	Total n(%)	Boys/men n(%)	Girls/women n(%)	***P***-value^**a**^ n(%)
**Toddlers**	112 (39.4)	55 (38.7)	57 (40.1)	0.808
**Children**	344 (82.7)	168 (79.2)	176 (86.3)	0.058
**Adolescents**	170 (79.8)	87 (81.3)	83 (78.3)	0.585
**Adults**	248 (89.9)	111 (89.5)	137 (90.1)	0.866
**Older adults**	242 (81.8)	114 (78.6)	128 (84.8)	0.171
**Lactating women**	135 (94.4)	–	135 (94.4)	0.139, 0.171^b^
**Pregnant women**	123 (92.5)	–	123 (92.5)	0.405, 0.484^b^

^a^*P-*values are based on chi-square tests

^b^*P*-values presented compared with adult men and adult non-lactating/non-pregnant women

Intakes of energy and all macronutrients, except fat, increased with age (except that protein was lower among older adults) and were mostly higher among boys/men than girls/women. Intakes of most micronutrients also increased with age, except calcium, which fluctuated among age groups. Most girls/women had higher intakes of vitamin A and C, whereas boys/men had higher intakes of vitamin B3, iron and sodium. Intakes of vitamin B1, B2 and calcium were sometimes higher among girls/women and sometimes among boys/men (Table 3).

### Energy intake

In all age groups, most of the boys/men and girls/women had an energy intake below the EER of active persons (Table 4), also below the EER for low-active and sedentary people (results not shown). Insufficient energy intake was detected in 39.4% of toddlers, 82.7% of children, 79.8% of adolescents, 89.9% of adults, 81.8% of older adults, 92.5% of pregnant women and 94.4% of lactating women. No significant differences were found between genders at all ages, nor between lactating/pregnant women and other adults.

**Table 3:** Characteristics of demographics, nutritional status and dietary intake in study population (*n* = 1771) by sex per age group

	Toddlers (***n*** = 284)		Children (***n*** = 416)		Adolescents (***n*** = 213)		Adult (***n*** = 276)		Older adults (***n*** = 296)		***Pregnant***	***Lactating***
	Boys (***n*** = 142)	Girls (n = 142)	Boys (*n* = 212)	Girls (***n*** = 204)	Boys (***n*** = 107)	Girls (***n*** = 106)	Men (*n* = 124)	Women (*n* = 152)	Men (*n* = 145)	Women (*n* = 151)	(*n* = 140)	(*n* = 146)
**Demographics**												
**Region**; n (%)												
- Northern-Lowland	44 (31.0)	49 (34.5)	67 (31.6)	75 (36.8)	41 (38.3)	30 (28.3)	41 (33.1)	59 (38.8)	48 (33.1)	47 (31.1)	46 (32.9)	51 (34.9)
- Central-SH	53 (37.3)	44 (31.0)	73 (34.4)	69 (33.8)	31 (29.0)	34 (31.2)	44 (35.4)	46 (30.3)	47 (32.4)	50 (33.1)	16 (32.9)	48 (32.9)
- Mekong Corridor	45 (31.7)	49 (34.5)	72 (34.0)	60 (29.4)	35 (32.7)	42 (39.6)	39 (31.5)	47 (30.9)	50 (34.5)	54 (35.8)	48 (34.2)	47 (32.2)
**Residence**; n (%)												
- Rural	105 (73.9)	103 (72.5)	155 (73.1)	153 (75.0)	79 (73.8)	75 (70.8)	91 (73.4)	110 (72.4)	106 (73.1)	110 (72.8)	103 (73.6)	106 (72.6)
- Urban	37 (26.1)	39 (27.5)	57 (26.9)	51 (25.0)	28 (26.2)	31 (29.2)	33 (26.6)	42 (27.6)	39 (26.9)	41 (27.2)	37 (26.4)	40 (27.4)
**Season**; n (%)												
- Rainy	72 (50.7)	67 (47.2)	113 (53.3)	103 (50.5)	50 (46.7)	58 (42.7)	63 (50.8)	77 (50.7)	71 (49.0)	77 (51.0)	66 (47.1)	72 (4.3)
- Dry	70 (49.3)	75 (52.8)	99 (46.7)	101 (49.5)	57 (53.3)	48 (45.3)	61 (492)	75 (49.3)	74 (51.0)	74 (49.0)	74 (52.9)	74 (50.7)
**Anthropometrics**												
**Age** (years); mean (SD)	1.8 (0.6)	1.8 (0.6)	5.1 (1.6)	5.1 (1.7)	12.4 (2.3)	12.6 (2.4)	37.8 (8.1)	34.4 (8.8)	62.1 (8.5)	60.7 (8.1)	26.7 (6.3)	26.8 (5.8)
**Weight** (kg); mean(SD)	10.5 (1.7)	10.2 (2.0)	16.8 (4.5)	16.3 (4.6)	36.1 (11.7)	37.7 (10.0)	60.9 (9.6)	55.2 (10.0)	57.2 (10.7)	53.7 (10.8)	57.3 (7.9)	52.8 (8.7)
**Height** (cm); mean(SD)	81.1 (6.6)	80.7 (8.3)	104.5 (11.8)	104.0 (11.9)	142.3 (13.8)	142.8 (10.5)	161.2 (5.4)	152.0 (4.9)	158.0 (6.0)	149.2 (5.3)	152.0 (5.1)	152.5 (5.4)
**BMI**; n (%)												
***P*-value**	0.14		0.69		**0.01**		146		0.08		**0.02**^**m**^	0.61^m^
											**0.01**^**w**^	0.06^w^
- Underweight	8 (5.6)	7 (4.9)	9 (4.2)	11 (5.4)	10 (9.3)	2 (1.9)	4 (3.2)	13 (8.6)	12 (8.3)	13 (8.6)	2 (1.4)	11 (7.5)
- Normal	113 (79.6)	118 (83.1)	185 (87.3)	178 (87.3)	86 (80.4)	93 (87.7)	59 (47.6)	59 (38.8)	66 (45.5)	48 (31.8)	44 (31.4)	77 (52.7)
- Overweight	18 (12.7)	9 (6.3)	13 (6.1)	13 (6.4)	2 (1.9)	7 (6.6)	26 (21.0)	28 (18.4)	27 (18.6)	31 (20.5)	38 (27.1)	26 (17.8)
- Obese	3 (2.1)	8 (5.6)	5 (2.4)	2 (1.0)	9 (8.4)	4 (3.8)	35 (28.2)	52 (34.2)	40 (27.6)	59 (39.1)	56 (40.0)	32 (21.9)
**Dietary intake;**												
median (IQR)												
**Energy (kcal)**	1040 (445)^a^	958 (386)^a^	1217 (425)^a^	1079 (421)^a^	1743 (681)	1490 (526)	1880 (650)	1517 (510)	1790 (638)	1434 (568)	1683 (571)	1681 (636)
**Macronutrients (g)**												
- Carbohydrates	171 (77)^a^	153 (67)^a^	206 (73)^a^	182 (71)^a^	326 (131)	264 (93)	338 (130)	276 (103)	320 (120)	268 (101)	305 (109)	312 (132)
- Protein	35 (17)^a^	33 (15)^a^	43 (19)^a^	39 (18)^a^	61 (29)	51 (22)	68 (28)	56 (22)	68 (30)	51 (25)	62 (16)	63 (26)
- Fat	21 (20)	21 (17)	23 (21)	19 (18)	21 (19)	20 (22)	19 (15)	16 (15)	19 (15)	13 (15)	20 (18)	17 (17)
- Dietary fiber	2.0 (2.8)	1.7 (2.3)	3.0 (2.7)	2.5 (2.5)	4.4 (3.2)	4.4 (3.4)	5.7 (3.5)	6.3 (4.3)	6.3 (4.4)	6.1 (5.2)	6.8 (5.3)	5.9 (5.9)

^a^ Dietary intake presented as mean (SD) instead of median (IQR)

^m^*P*-values are based on Chi-square tests compared to adult men; ^w^*P*-values are based on Chi-square tests compared to adult women

*P*-values are based on Chi-square tests

**Table 5:** Differences in imbalance nutrient intake of macro- and micronutrients by sexes per age group

	Toddlers (n = 284)			Children (n = 416)			Adolescents (n = 213)			Adult (n = 276)			Older adults (n = 296)			Pregnant women (n = 140)			Lactating women (n = 146)		
	Boys (*n* = 142)	Girls (*n* = 142)		Boys (*n* = 212)	Girls (*n* = 204)		Boys (*n* = 107)	Girls (*n* = 106)		Men (*n* = 124)	Women (*n* = 152)		Men (*n* = 145)	Women (*n* = 151)							
	*n* (%)	*n* (%)	*p*^a^	*n* (%)	*n* (%)	*P*^a^	*n* (%)	*n* (%)	*P*^*a*^	*n* (%)	*n* (%)	*P*^*a*^	*n* (%)	*n* (%)	*P*^*a*^	*n* (%)	*P*^b^	*P*^c^	*n* (%)	*P*^b^	*P*^c^
**Macronutrients**																					
Carbohydrates	23 (16.2)	29 (20.4)	0.357	7 (3.3)	18 (8.8)	**0.018**	1 (0.9)	3 (2.8)	0.369^f^	0 (0.0)	1 (0.7)	1.000^f^	1 (0.7)	4 (2.6)	0.371^f^	8 (5.7)	**0.008**^**f**^	**0.016**^**f**^	16 (11.0)	**< 0.0001**	**< 0.0001**
Protein	4 (2.8)	6 (4.2)	0.52	1 (0.5)	4 (2.0)	0.208^f^	5 (4.7)	9 (8.5)	0.261	10 (8.1)	27 (17.8)	**0.019**	20 (13.8)	47 (31.1)	**< 0.0001**	43 (31.9)	**< 0.0001**	**0.006**	60 (41.1)	**< 0.0001**	**< 0.0001**
Fat	ND	ND	–	ND	ND	–	ND	ND	–	ND	ND	–	ND	ND	–	ND	–	–	ND	–	–
Dietary fibre^g^	142 (100)	142 (100)	NA	211 (99.5)	204 (100)	1.000^f^	107 (100)	105 (99.1)	0.314	124 (100)	152 (100)	NA	143 (98.6)	149 (98.7)	1.000^f^	139 (99.3)	1.000^f^	0.479^f^	146 (100)	NA	NA
**Vitamins**																					
A																					
- nsufficient intake^d^	89 (62.7)	73 (51.4)	0.073	177 (83.5)	164 (80.4)	0.66	104 (97.2)	96 (90.6)	**0.043**	119 (96.0)	139 (91.4)	0.13	127 (87.6)	142 (94.0)	0.054	123 (87.9)	**0.017**	0.479	141 (96.6)	1.000^f^	0.063
- Excessive intake^e^	16 (11.3)	14 (9.9)		12 (5.7)	12 (5.9)		0 (0.0)	3 (2.8)		0 (0.0)	5 (3.3)		5 (3.4)	2 (1.3)		4 (2.9)			3 (2.1)		
B1 (Thiamin)^h^																					
- Insufficient intake^d^	97 (68.3)	91 (64.1)	0.452	149 (70.3)	146 (71.6)	0.773	85 (79.4)	83 (78.3)	0.839	105 (84.7)	106 (69.7)	**0.004**	115 (79.3)	117 (77.5)	0.703	119 (85.0)	0.942	***0.002***	128 (87.7)	0.476	**< 0.0001**
B2 (Riboflavin)^h^																					
- Insufficient intake^d^	71 (50.0)	59 (41.5)	0.153	124 (58.5)	123 (60.3)	0.708	88 (82.2)	82 (77.4)	0.375	108 (87.1)	122 (80.3)	0.13	124 (85.5)	132 (87.4)	0.633	112 (80.0)	0.123	0.955	132 (90.4)	0.388	**0.014**
B3 (Niacin)																					
- Insufficient intake^d^	89 (62.7)	88 (62.0)	0.955	108 (51.0)	112 (54.9)	0.527	62 (57.9)	66 (62.3)	0.52	57 (46.0)	88 (57.9)	**0.048**	60 (41.4)	99 (65.6)	**< 0.0001**	118 (84.3)	**< 0.0001**	**< 0.0001**	123 (84.2)	**< 0.0001**	**< 0.0001**
- Excessive intake^e^	14 (9.9)	13 (9.2)		6 (2.8)	8 (3.9)		5 (4.7)	1 (0.9)		2 (1.6)	0 (0.0)		2 (1.4)	0 (0.0)		1 (0.7)			1 (0.7)		
C																					
- Insufficient intake^d^	80 (56.3)	73 (51.4)	0.405	152 (71.7)	149 (73.0)	0.76	77 (72.0)	81 (76.4)	0.458	102 (82.3)	105 (69.1)	**0.011**	102 (70.3)	95 (62.9)		96 (68.6)			125 (85.6)		
- Excessive intake^e^	0 (0.0)	0 (0.0)		0 (0.0)	0 (0.0)		0 (0.0)	0 (0.0)		0 (0.0)	0 (0.0)		0 (0.0)	0 (0.0)	0.176	0 (0.0)	**0.01**	0.925	0 (0.0)	0.452	**0.001**
**Minerals**																					
Calcium^g^																					
- Insufficient intake^d^	106 (74.7)	90 (63.4)	0.04	206 (97.2)	197 (96.6)	0.725	104 (97.2)	105 (99.1)	0.621^f^	119 (96.0)	152 (100)	NA	142 (97.9)	149 (98.7)	NA	138 (98.6)	0.259^f^	0.229^f^	142 (97.3)	0.736	**0.04**
- Excessive intake^e^	3 (2.1)	0 (0.0)		0 (0.0)	0 (0.0)		0 (0.0)	0 (0.0)		1 (0.8)	0 (0.0)		0 (0.0)	1 (0.7)		0 (0.0)			0 (0.0)		
Iron																					
- Insufficient intake^d^	53 (37.3)	59 (41.5)	0.466	88 (41.5)	99 (48.5)	0.15	66 (61.7)	69 (65.1)	0.605	36 (29.0)	94 (61.8)	< 0.0001	41 (28.3)	57 (37.7)	0.083	128 (91.4)	**< 0.0001**	**< 0.0001**	66 (45.2)	**0.006**	**0.004**
- Excessive intake^e^	0 (0.0)	0 (0.0)		0 (0.0)	0 (0.0)		0 (0.0)	0 (0.0)		0 (0.0)	1 (0.7)		0 (0.0)	0 (0.0)		0 (0.0)			1 (0.7)		
Sodium^g^																					
- Insufficient intake^d^	59 (41.5)	52 (36.6)	0.611	66 (31.1)	67 (32.8)	0.132	15 (14.0)	17 (16.0)	0.916	14 (11.3)	21 (13.8)	0.746	14 (9.7)	26 (17.2)	**0.004**	23 (16.4)	0.469	0.812	17 (11.6)	0.916	0.578
- Excessive intake^e^	61 (43.0)	63 (44.4)		87 (41.1)	97 (47.6)		73 (68.2)	71 (67.0)		89 (71.8)	103 (67.8)		110 (75.9)	87 (57.6)		93 (66.4)			107 (73.3)		

Inadequate intake was defined as insufficient intake (for the macronutrients and vitamin B1 and B2) or, when an Tolerable Upper Intake Level (UL) was available, as insufficient or excessive intake (for all other vitamins and minerals). Insufficient intake: participants who had intakes below the Estimated Average Requirement (EAR) or Adequate Intake (AI) for the nutrient of interest. Excessive intake: participants who had an intake that exceeded the UL for the nutrient of interest. ND = No data on EAR or AI**.** NA = Chi-Square not applicable, as among both men and women 100% did not meet the EAR/AI requirements (for dietary fiber) or > 20% of the cells has expected count less than 5 and merging categories together (sufficient intake and excess intake) did not solve this ‘problem’ (for calcium)

^a^*P*-values are based on Chi-square tests

^b^*P*-values are based on Chi-square tests compared with adult men

^c^*P*-values are based on Chi-square tests compared with adult (non-lactating) women

^d^Insufficient intake was defined as intake of the nutrient of interest below the recommended EAR or AI

^e^Excess intake as intake of the nutrient of interest above the UL

^f^ Data were calculated with Fisher’s Exact Test instead of Chi-square test

^g^Adequate Intake (AI) used as DRI instead of EAR

^h^No Tolerable Upper Intake Level (UL) has been established

**Table 6:** Association between demographics, BMI, nutritional status and inadequate micronutrient intake

	Adolescents						Adult						Pregnant women			Lactating women		
	**Inadequate**	**OR (95%-CI)**	***P*-values**	**Inadequate**	**OR (95%-CI)**	***P*-value**	**Inadequate**	**OR (95%-CI)**	***P*-value**	**Inadequate**	**OR (95%-CI)**	***P*-value**	**Inadequate**	**OR (95%-CI)**	***P*-value**	**Inadequate**	**OR (95%-CI)**	***P*-value**
	Boys, Freq (%)			Girls, Freq (%)			Men, Freq (%)			Women, Freq (%)			Subject, Freq (%)			Subject, Freq (%)		
**Residence**																		
- Rural	74 (93.7)	REF		64 (85.3)	REF	0.179	83 (91.2)	REF		96 (87.3)	REF		95 (92.2)	REF		99 (93.4)	REF	
- Urban	21 (75.0)	0.20 (0.06–0.71)	**0.012**	23 (74.2)	0.94 (0.18–1.38)		29 (87.9)	0.70 (0.20–2.50)	0.581	34 (81.0)	0.62 (0.24–1.61)	0.325	33 (89.2)	0.70 (0.20–2.46)	0.572	37 (92.5)	0.87 (0.21–3.55)	0.848
**Season**						0.759												
- Rainy	41 (82.0)	REF		47 (81.0)	REF		54 (85.7)	REF		61 (79.2)	REF		61 (92.4)	REF		68 (94.4)	REF	
- Dry	54 (94.7)	3.95 (1.01–15.52)	**0.049**	40 (83.3)	1.17 (0.43–3.19)		58 (95.1)	3.22 (0.83–12.53)	**0.091**	69 (92.0)	3.02 (1.11–8.20)	**0.03**	67 (90.5)	0.79 (0.24–2.60)	0.692	68 (91.9)	0.67 (0.18–2.47)	0.544
**Region**																		
- Northern-Lowland	40 (97.6)	REF		26 (86.7)	REF		40 (97.6)	REF		54 (91.5)	REF		45 (97.8)	REF		47 (92.2)	REF	
- Central-SH	26 (83.9)	0.13 (0.01–1.18)	0.07	26 (76.5)	0.50 (0.13–1.87)	0.303	40 (90.9)	0.25 (0.03–2.34)	0.224	39 (84.8)	0.52 (0.15–1.75)	0.287	44 (95.7)	0.49 (0.04–5.59)	0.565	45 (93.8)	1.28 (0.27–6.03)	0.758
- Mekong Corridor	29 (82.9)	0.21 (0.01–1.06)	0.056	35 (83.3)	0.77 (0.20–2.91)	0.699	32 (82.1)	0.11 (0.01–0.98)	**0.048**	37 (78.7)	0.34 (0.11–1.08)	**0.068**	39 (81.3)	0.10 (0.01–0.79)	**0.03**	44 (93.6)	1.25 (0.26–5.90)	0.78
**BMI**																		
- Underweight	10 (100.0)	NA	0.999	2 (110.0)	NA	0.999	4 (100.0)	NA	0.999	11 (84.6)	0.86 (0.16–4.63)	0.863	2 (100.0)	NA	0.999	11 (100.0)	NA	0.999
- Normal weight	79 (91.9)	REF		76 (81.7)	REF		53 (89.8)	REF		51 (86.4)	REF		40 (90.9)	REF		73 (94.8)	REF	
- Overweight	0 (0.0)	0.0 (0.0–0.0)	0.999	5 (71.4)	0.56 (0.10–3.13)	0.508	23 (88.5)	0.87 (0.20–3.77)	0.85	23 (82.1)	0.72 (0.21–2.45)	0.6	37 (97.4)	3.70 (0.40–34.63)	0.252	24 (92.3)	0.66 (0.11–3.82)	0.64
- Obesity	6 (66.7)	0.18 (0.04–0.87)	**0.033**	4 (100.0)	NA	0.999	32 (91.4)	1.21 (0.28–5.17)	0.799	45 (86.5)	1.01 (0.34–3.00)	0.988	49 (87.5)	0.70 (0.19–2.56)	0.59	28 (87.5)	0.38 (0.09–1.64)	0.196

Inadequate micronutrient adequacy as < 4 adequate micronutrient intakes (out of 8 in total). Reference category = inadequate micronutrient adequacy, defined as < 4 adequate micronutrient intakes.

*NA* Not applicable, *REF* Reference category, *REF* Reference category, *OR* Odd Ratio

*P*-value = Level of significance

### Acceptable macronutrient distribution ranges

The majority of men and women in all age groups, except female toddlers (42.3%), had an energy intake from carbohydrates above the AMDR, and an energy intake from fat below the AMDR. The energy intake from protein fell within the range of the AMDR for the majority of boys/men and girls/women of all age groups (ranging from 88.8 to 97.9%). The only significant differences in insufficient intakes related to AMDRs between boys/men and girls/women were for carbohydrates among toddlers (*p =* 0.030) and older adults (*p =* 0.013). Pregnant and lactating women had greater non-adherence to the AMDR of fat compared to adult men (respectively *p =* 0.034, and *p =* 0.011).

### Macro- and micronutrients intake

**Fig. 1:**
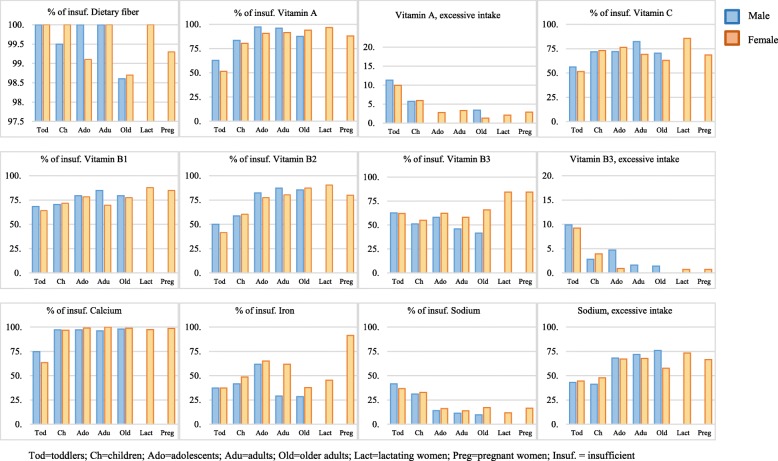
Proportions (%) of insufficient intake and excessive nutrients per age group and gender

Nearly all subjects had critically insufficient intakes of dietary fiber, while more than half had an insufficient intake of vitamins A, C, B1 and calcium. Insufficient intake of iron was high predominantly among adolescent boys (61.7%) and girls (65.1%), adult women (61.8%), and most particularly, pregnant women (91.4%). Excessive sodium intake was found in all age groups and both genders, with more than 40% of the study population, especially starting from adolescence. The only major macronutrient insufficiencies were found for protein among both older adults and lactating and pregnant women (Fig. 1).

### Associations between micronutrients intake and gender

Comparing between genders, women had higher prevalence of insufficient nutrient intakes than men in almost all age groups for almost all nutrients. We found significantly more carbohydrate insufficiency among girl children (8.8%) than boys (3.3%) (*p =* 0.01). Inadequate intake of protein was more prevalent among adult women (17.8%), older adult women (31.1%), pregnant women (31.9%), and lactating women (41.1%) compared to men and other women (*p* < 0.01). Insufficient intake of vitamin B3 was more common among adult women (57.9%), older women (65.6%), pregnant women (84.3%) and lactating women (84.2%) than among men and other women in the same age groups. Insufficient intake of iron was also significantly more frequent in adult women (61.8%), pregnant women (45.2%) and lactating women (91.4%), compared to men and other women in the same age groups.

For men, insufficient intake of vitamin A was more common among adolescent boys (97.2%) than girls (90.6%). We also found a difference between pregnant women (87.9%) and men (*p* < 0.04). Intake of vitamin B1 was more often insufficient among adult men (84.7%) than women in the same age group (69.7%). Among toddlers, there was a significant difference in calcium intake between boys (74.7%) and girls (63.4%) (Table 5).

### Association between demographic features, nutritional status, and inadequate micronutrient

Nine significant associations between BMI or demographics and insufficient micronutrient intake among the different age and gender groups were found. It was notable that adolescent boys accounted for three significant associations; they had low probability to have micronutrient insufficiency among obese boys who live in the Mekong corridor, but in the dry season, increased probability of inadequate micronutrient intake. Living in the Mekong corridor was associated with significantly less probability of insufficient intake of micronutrients among toddlers, adolescent girls, pregnant woman, and men, compared with other regions. Children and adolescent boys residing in an urban area had significantly lower probability of insufficient intake of micronutrients than those living in rural areas. Again, among children and adolescent boys, there was more probability of inadequate micronutrient intake during the dry season than in the rainy season. Living in the Northern-Lowland region was associated with a higher probability of inadequate micronutrient intake in males, from toddler to adult ages. No associated difference was found in the older adult group for both sexes. In the table we have not shown the toddler, children and older adult groups where no significant differences were found. (Table 6).

## Discussion

The aim of this study was to determine the influence of gender and age on dietary intake and BMI with a particular focus on possible gender disparities, for the first time in Lao PDR. These new insights may be used to optimize current and to design new nutritional interventions that aim to improve dietary intakes of the population of Lao PDR.

The most obvious gender disparities were particularly found between pregnant or lactating women and other groups of adults (adult men, non-lactating, non-pregnant women). Those women had significantly higher prevalence of insufficient intakes of carbohydrates, proteins, vitamin B1 (only lactating), B2, B3, C (only lactating) and iron (only pregnant). Earlier studies also reported higher rates of (multiple) micronutrient deficiencies during pregnancy and lactation, especially in Southeast Asian LMICs [19, 27]. That pregnant and lactating women had insufficient intakes in nutrients may possibly be due to personal beliefs and traditional practices around pregnancy and lactation in Lao PDR. During pregnancy it is common to eat less and to avoid certain foods, such as fruit, raw vegetables, pork and spicy foods [17, 28]. Also, after birth, Lao women often follow a restricted diet for up to 40 days after giving birth, eating small amounts of food containing little to no nutritional value.

The most important differences were that insufficient intakes of protein, vitamin B3 and iron were more prevalent among women than men. These findings match those of previous studies in other LMICs that found dietary intakes to be largely similar by sex, but where gender differences do exist, it is usually women who have the lower nutrient intakes [29, 30]. Socially- and culturally-constructed roles related to food might explain why gender differences were mainly found among adults and not children, and why women were particularly more affected. Men are often allowed to eat first [20], and women, as primary caregivers and food providers, often feed their children and husbands before themselves [11]. However, for some nutrients, the prevalence of insufficient intakes was higher among boys and men, such as for vitamin B1 and B2. A possible explanation for this may be that national nutrition interventions mainly focus on children, adolescent girls, and women of reproductive age [31]. An interesting finding was that insufficient intake of calcium was seriously high for all subjects, at nearly 100% of the population, and with no difference between genders except among toddlers, where insufficiency was slightly higher among boys. In this life stage breastfeeding and breast milk substitute are key sources of calcium as most children are still breastfeeding [32]. Due to lack of data on breastfeeding this explanation could not be investigated.

There are several possible explanations as to why only a few gender differences were found. Firstly, diets of Lao people do not vary much by sex and age most of the time; only during special periods like pregnancy and lactation is food intake dependent on local beliefs and demographic characteristics [18]. The main diet for everyone is characterized by white sticky rice as the staple food and a low intake of fruits and vegetables [27]. Such diets provide little dietary fiber and micronutrients; fruits and vegetables are an important source of these nutrients and traditional rice processing practices destroy the water soluble vitamins in sticky rice [33]. Secondly, it might be possible that men and women of the same household for convenience reported the same dietary intake with limited portion size difference. However, data collection teams were trained to avoid this repetition where possible.

We also found that boys/men had higher weight and height than girls/women in almost all age groups, but BMI showed that the prevalence of overweight and obesity were high starting from adulthood in both sexes. If we compared between genders, we found overweight and obesity were higher among boy toddlers and children compared to girls, but during adolescence, boys had significantly higher prevalence of underweight and obesity than girls. The association of these conditions with demographics also showed the effect in adolescent boys. The theory is that males have higher body weight, height, and higher BMR; therefore higher nutrient requirement, especially during the growth period of life [13]. From adolescents through adulthood into older age, girls/women had a higher prevalence of overnutrition than boys/men. Overweight and obesity are considered a public health problem. Our findings support a previous study indicating that prevalence of male and female obesity has continued to grow in developing countries like Laos since 1999 [4, 33]. Our findings also confirm that the double burden of malnutrition has now appeared in our country, supporting previous findings on dietary intake in Lao PDR [34, 35]. For example, a previous cross-sectional study among Lao mothers showed insufficient intake of vitamin A, iron and calcium among more than 90% of mothers [36]. The major insufficient intakes of vitamin A found in our study are consistent with previous studies [35, 36], however controversy still exists, as other studies have reported vitamin A intake as sufficient [34]. Our finding of inadequate intakes of vitamin B1 and B2 among both sexes was also found in previous studies in Lao PDR [26]. The insufficient intake of specific micronutrients such as vitamin A, vitamin B1 or vitamin C are known from clinical evidence to be related to obesity and metabolic syndrome [37]; we found both genders to have a high prevalence of obesity and insufficient intakes of vitamin A, B1 and C, especially in adult women. Further research and intervention studies need to considered, based on this information, and focusing on both under-nutrition and over-nutrition for all life stages of both men and women.

The intake of most nutrients is often strongly associated with energy intake. In general, intake of micronutrients is lower when energy intake is low [38]. This was also seen in our study. Energy intake of the majority of participants was below the EER of active persons. This was an unexpected finding for a population including normal to overweight and obesity. However, under-reporting of energy intake from self-reported 24hDRs is common, with reported intakes beginning 50–110% lower than actual intakes [39, 40]. This might also be the case in our study. Energy intake was not statistically adjusted, since it has been criticized as being inadequate to control for reported energy intake [41–43].

Most of the study population had a dietary imbalance, high in carbohydrate and low in fat. Studies implemented in Vietnam and neighboring countries found similar results of an imbalanced diet [44]. Since fat provides more energy (9 cal per gram compared to 4 for carbohydrate and protein), low fat intake might have contributed to the observed insufficient energy intakes and to deficiency of fat-soluble vitamins. Although there is no EAR for fat, it can be assumed that fat intake is insufficient among all age groups since the energy intake from fat was far below the AMDR. This is worrisome as inadequate fat intake may lead to impaired growth and increased risk of chronic diseases. Particularly children and pregnant women are vulnerable to inadequate fat intake as fat requirements are relatively high for (fetal) development [22]. However, to promote the fat and to recommend that the population change eating behavior towards a higher fat intake needs careful consideration, in terms of the type of fatty acid and its portion for each life stage. Otherwise the changes could result in unhealthy weight gains and negative effects in long-term diseases [45] because we also found overweight and obesity to be one of the burdens of malnutrition in Lao PDR. Overall, protein intake was sufficient, except that there were relatively high proportions of insufficient intake among older adult women (31.1%), and pregnant women (31.9%) and lactating women (41.1%). Precisely during these life stages, getting enough protein requires more attention since it is required for optimal maternal and child health and for maintenance of muscle function in the elderly [46, 47].

An interesting finding was that excessive intake of sodium was more prevalent than insufficient intake. Excessive sodium intake increases the risk of hypertension, which was already prevalent among 20% of adults in Lao PDR in 2013 [32]. This is worrisome as hypertension can lead to serious health problems as cardiovascular diseases and chronic renal failure [48].

Micronutrient intake was insufficient among the majority of participants in our study; the only significant difference was for lactating women, who were more likely to have micronutrient inadequacies than other women; this is consistent with a previous finding which found that multiple micronutrient intakes of lactating women were below the standard of EAR [49]. In our study, living in rural areas, consumption during dry season, and living in the Northern-Lowland region were associated with inadequate micronutrient intakes among men and women of certain age groups only. The association with rural areas might be explained by findings that rural residents in Lao PDR have relatively less balanced meals than do urban residents [31]. Recent studies also reported the association of dry season with lower intakes of energy and most micronutrients [20, 50, 51]. Since there is also a harvest of irrigated rice in April [52], which falls in the middle of the dry season, the association between dry season and insufficient dietary intake might not have been found among all ages. No previous studies investigated the association of region with micronutrient adequacy in Lao PDR, so our findings could not be placed in context. It should be noted that we had to define micronutrient adequacy for this study, because no set measures of micronutrient adequacy of diets based on actual micronutrient intakes are available. We recommend further investigating the application of such scores. They might be useful in addition to, or as a substitute for, proxy measures of nutrient quality of diets, based on the number of consumed food groups such as the dietary diversity score, which is not a true measure of nutritional adequacy as it disregards food intake [53, 54].

To our knowledge, this is the first study on a substantial study population that investigated dietary intakes in Lao PDR with a focus on gender differences. The study sample was representative for the population of Lao PDR, as participants of all age groups (toddlers up to and including older adults) were included, and men and women were more or less equally represented. The sample size of each age group was calculated, planned and adapted in the field, because the available sample was not always what was planned; some age groups were slightly higher while others were slightly lower. Also, by presenting results per age group and gender, including pregnant and lactating women, specific dietary needs and requirements for various life stages were taken into account. This enables us make recommendations tailored to the needs of every individual. Moreover, dietary intakes of energy, all macronutrients and eight micronutrients were examined, which provides a clear indication of overall dietary intake of the Lao population. Lastly, to minimize seasonal effects of dietary intake (e.g. changes in quantity and quality of consumed foods), 24hDRs were carried out twice, once in the rainy season and once in the dry season.

This study also had limitations. Firstly, the 24hDR relies heavily on a person’s memory which may have led to recall bias and/or giving socially desirable responses, usually resulting in an underestimation of the actual intake [55]. Moreover, the 24hDR only provides a snapshot of dietary intake of one specific day. An individual’s intake may vary largely from day to day, resulting in an inaccurate estimate of the (long-term) usual intake based on that 1 day [55]. We did repeat the study in the dry and the rainy seasons, to accommodate the differences between these two times of the year. The limitations of the 24hDR, however, probably should not bias the examination of gender differences, which was the aim of this study. Another limitation is that no Lao/Asian DRIs were used, as no national dietary guidelines exist and the most recent Southeast Asian guidelines are incomplete, have not been revised since 2008 [9]. Since the obsolete Southeast Asian DRIs were generally lower, the use of Caucasian DRIs may have led to incorrect estimates (most likely overestimates) of insufficient intakes in this study. The results therefore need to be interpreted with caution. Thirdly, although the sampling method was random, residents of very remote rural areas were not included. The prevalence of insufficiencies among these residents is suspected to be even higher, since food security and diets are reported to be very poor in remote rural areas [56]. We did not have data on socio-demographic characteristics, such as ethnicity, education, and occupation, which might also be associated with dietary intake.

Overall, dietary intake of a large sample of people from children to older adults in Lao PDR was alarmingly insufficient. Gender differences were, however, minimal. Intakes of energy, fat, dietary fiber and all micronutrients, except sodium, were highly insufficient among all study participants. Pregnant and lactating women were disproportionately affected by insufficiencies. The prevalence of overweight and obesity were also high in both sexes, especially in women in adult to older age; this is often referred to as the double burden of malnutrition and is a public health problem. The findings of this study emphasize the importance of nutritional interventions targeting all Lao people. Policy makers should focus on evaluating and modifying current nutritional interventions and developing new ones, focusing not only on children and women of reproductive age, but also on men and women and on both undernutrition and overnutrition. Tailored interventions are needed to achieve and maintain good nutritional health throughout the lifespan, to prevent adverse health outcomes, and to reduce the prevalence of FNI and the DBM [57, 58]. Multiple nutritional issues should be addressed, including increasing fat but in careful consideration of type and portion; increasing micronutrient intake among all, as well as focus on deficiencies found in each life stage; increasing protein intake during pregnancy, lactation and old age; and promoting reduction in sodium intake.

Given that nutrient intakes are related to dietary pattern and consumed food groups [54], further research towards intake of food groups in term of food choice, food preference and the relation with nutrient intake among Lao men and women and its association with nutritional status is recommended. A more detailed investigation of possible gender differences in all of these would also be helpful. More data is needed on the diets of people living in remote rural areas. Socio-demographic information such as ethnicity and education should be included in future studies to identify whether any are associated with issues related to dietary intake. Better estimates of energy intake could be gained by collecting data on individual physical activity levels and EER. Lastly, in further research on gender differences on dietary intake in Lao PDR, it is recommended to adjust for daily observed intakes to estimate usual intakes of the study population as that would provide a better estimation of the proportion of individuals with insufficient intakes.

## Conclusion

The results of our study demonstrated that a large proportion of people in Lao PDR have insufficiencies in the amount and quality of food they consume. Pregnant and lactating women were at increased disadvantage with more severely insufficient intakes than others. Adolescent boys were also shown to have certain insufficient intakes in higher proportions than girls. The issues of macronutrient imbalance and double burden malnutrition were confirmed for Lao PDR.

## Supplementary information

**Additional file 1.** Appendix 1

**Additional file 2.** Appendix 2

## Data Availability

Data will be available from the corresponding author upon request.

## Abbreviations

24hDR: 24-h dietary recall
AI: Adequate intake
AMDRs: Acceptable macronutrient distribution ranges
BMI: Body mass index
BMR: Basal metabolic rate
DBM: Double burden” of malnutrition
DRIs: Dietary reference intakes
EAR: Estimated average requirement
EER: Estimated energy requirement
FAO: Food and Agriculture Organization
FCS: Food consumption survey
FNI: Food and nutrition insecurity
IQR: interquartile range
Lao PDR: Lao People’s Democratic Republic
LMICs: Low- and middle-income countries
LTPHI: Lao Tropical and Public Health Institute
MOH: Ministry of Health
OR: Odds ratio
PAL: Physical activity level
RDA: Recommended dietary allowance
REF: Reference
SD: Standard deviation
UL: Tolerable upper intake level
WHO: World Health Organization
