# Supplementary material for: Are dietary intake and nutritional status influenced by gender? The pattern of dietary intake in Lao PDR: a developing country
Source: Nutr J. 2020 Apr 11;19:31. doi: 10.1186/s12937-020-00545-9 (PMC7151640; doi:10.1186/s12937-020-00545-9)
Supplement: Supplementary file 1 — Additional file 1. Appendix 1 [file 12937_2020_545_MOESM1_ESM.docx]

# **Appendix1**

**ID**

**Questionnaires form for 24 hour recall**

**Target population**: 1. Sample 2. Sub sample

**Season**: 1. Raining 2. Dry

**Person who provide information**

1. Care taker 2. Care taker and self 3. Self . Name of person who give information.............................age..............

**Date of interview**.................................................... **Name of sample**…………..............….......**surname**..….……......… . **Sex** 1. Male 2. Female **age**.........year .........month

**Target group**

1. Infants (3 to 11 months) 2. Toddler (12 to 36 months) 3. Children (3 to 5.9 years)

4. Adolescent (6 to 14.9 years) 5. Young Adults (15 to 45 years) 6. Elderly (> 50 years)

7. Pregnant women……………..month 6. Lactating women (age of child ……..year……month)

Food cosumed yeat erday: 1. Normal day 2. Holiday Pattern of food consumes 1. General 2.Special 3. Ill

Quantity of food consumes yesterday compared with other day 1. Less then 2. Equal 3. More then

**Time to start**...................... **Time to complet**.......................

**Name of interviewer** .………………………………

| **Time** | **Meal** | **Source** | **Menu** | **Total amount consumes** | **Ingredients** | **Amount of ingredients consumes** | **Note** | **Code**  **For staff** |
| --- | --- | --- | --- | --- | --- | --- | --- | --- |
|  |  |  |  |  |  |  |  |  |
|  |  |  |  |  |  |  |  |  |
|  |  |  |  |  |  |  |  |  |
|  |  |  |  |  |  |  |  |  |
|  |  |  |  |  |  |  |  |  |
|  |  |  |  |  |  |  |  |  |
|  |  |  |  |  |  |  |  |  |
|  |  |  |  |  |  |  |  |  |
|  |  |  |  |  |  |  |  |  |
|  |  |  |  |  |  |  |  |  |
|  |  |  |  |  |  |  |  |  |
|  |  |  |  |  |  |  |  |  |
|  |  |  |  |  |  |  |  |  |
|  |  |  |  |  |  |  |  |  |
|  |  |  |  |  |  |  |  |  |
|  |  |  |  |  |  |  |  |  |
|  |  |  |  |  |  |  |  |  |
|  |  |  |  |  |  |  |  |  |
|  |  |  |  |  |  |  |  |  |
|  |  |  |  |  |  |  |  |  |
|  |  |  |  |  |  |  |  |  |
|  |  |  |  |  |  |  |  |  |
|  |  |  |  |  |  |  |  |  |
| **Time** | **Meal** | **Source** | **Menu** | **Total amount consumes** | **Ingredients** | **Amount of ingredients consumes** | **Note** | **Code**  **For staff** |
|  |  |  |  |  |  |  |  |  |
|  |  |  |  |  |  |  |  |  |
|  |  |  |  |  |  |  |  |  |
|  |  |  |  |  |  |  |  |  |
|  |  |  |  |  |  |  |  |  |
|  |  |  |  |  |  |  |  |  |
|  |  |  |  |  |  |  |  |  |
|  |  |  |  |  |  |  |  |  |
|  |  |  |  |  |  |  |  |  |
|  |  |  |  |  |  |  |  |  |
|  |  |  |  |  |  |  |  |  |
|  |  |  |  |  |  |  |  |  |
|  |  |  |  |  |  |  |  |  |
|  |  |  |  |  |  |  |  |  |
|  |  |  |  |  |  |  |  |  |
|  |  |  |  |  |  |  |  |  |
|  |  |  |  |  |  |  |  |  |
|  |  |  |  |  |  |  |  |  |
|  |  |  |  |  |  |  |  |  |
|  |  |  |  |  |  |  |  |  |
|  |  |  |  |  |  |  |  |  |
|  |  |  |  |  |  |  |  |  |
