# Supplementary material for: Are dietary intake and nutritional status influenced by gender? The pattern of dietary intake in Lao PDR: a developing country
Source: Nutr J. 2020 Apr 11;19:31. doi: 10.1186/s12937-020-00545-9 (PMC7151640; doi:10.1186/s12937-020-00545-9)
Supplement: Supplementary file 2 — Additional file 2. Appendix 2 [file 12937_2020_545_MOESM2_ESM.docx]

**Appendix 2**

**Table X1**. Equations to estimate the Estimated Energy Requirement (EER) per individual.

|  | **EER (kcal/day)** |
| --- | --- |
|  | *Total Energy Expenditure + Energy Deposition* |
| **Toddlers** | (89 x weight [kg] –100) + 20 |
| **Children**  - Boys  - Girls | 88.5 – (61.9 x age [y]) + PA x [(26.7 x weight [kg]) + (903 x height [m])] + 20  135.3 – (30.8 x age [y]) + PA x [(10.0 x weight [kg]) + (934 x height [m])] + 20 |
| **Adolescents**  - Boys  - Girls | 88.5 – (61.9 x age [y]) + PA x [(26.7 x weight [kg]) + (903 x height [m])] + 25  135.3 – (30.8 x age [y]) + PA x [(10.0 x weight [kg]) + (934 x height [m])] + 25 |
|  | *Total Energy Expenditure* |
| **Adults**  - Men  - Women | 662 – (9.53 x age [y]) + PA x [(15.91 x weight [kg]) + (539.6 x height [m])]  354 – (6.91 x age [y]) + PA x [(9.36 x weight [kg]) + (726 x height [m])] |
| **Older adults**  - Men  - Women | 662 – (9.53 x age [y]) + PA x [(15.91 x weight [kg]) + (539.6 x height [m])]  354 – (6.91 x age [y]) + PA x [(9.36 x weight [kg]) + (726 x height [m])] |
|  | *Nonpregnant EER + Milk Energy Output – Weight Loss* |
| **Lactating women**  - 0-6 m postpartum  - 7-12 m postpartum | Non-pregnant EER + 500 – 170  Non-pregnant EER + 400 – 0 |
|  | *Nonpregnant EER + Pregnancy Energy Deposition* |
| **Pregnant women**  - 1^st^ trimester  - 2^nd^ trimester  - 3^rd^ trimester | Non-pregnant EER + 0  Non-pregnant EER + 340  Non-pregnant EER + 452 |

EER = Estimated Energy Requirement, PA = Physical Activity Level

The actual energy intake of the participants was used to compared to the Estimate Energy Requirement (EER). The EER is based on gender, age, weight, height and a physical activity level (PAL), and therefore individually calculated for each participant by prediction equations (**Table X1**). Since most Lao people work in the agricultural sector (24) and an active PAL is recommended to maintain health (23), this PAL was used in the equations to estimate EERs.

Intakes of carbohydrate, protein and fat were assessed relative to another, as they can substitute for another in order to meet the energy requirement (23), and compared with Acceptable Macronutrient Distribution Ranges (AMDRs), which are expressed as percentage of total energy intake. Lastly, intakes of macro- and micronutrients were compared to the Estimated Average Requirement (EAR), as it is the most appropriate Dietary Reference Intakes (DRI) to assess group nutrient intakes to estimate the prevalence of (in)sufficiency. For nutrients without an EAR, the Adequate Intake (AI) was used instead. For micronutrients with a Tolerable Upper Intake Level (UL) as vitamin A, B3, C, calcium, iron and sodium, excessive intake was also assessed. The EARs/AIs and ULs of men and women per age group are summarized in **Table X2**. Intakes of participants that met the EER for energy, the AMDR for carbohydrate, protein and fat, and the EAR/AI for macro- and micronutrients were defined as ‘sufficient’, intakes that did not as ‘insufficient’, and intakes that exceeded the UL as ‘excessive’, in order to assess population dietary (in)sufficiency compared to the DRIs.

**Table X2** Dietary Recommended Intakes (DRIs), presented as EAR (UL) by sex per age group.

|  | **Macronutrients (g/day)** | | | | **Micronutrients** | | | | | | | |
| --- | --- | --- | --- | --- | --- | --- | --- | --- | --- | --- | --- | --- |
|  |  | | | | **Vitamins (mg/day)** | | | | | **Minerals (mg/day)** | | |
|  | *Carbs* | *Protein^a^* | *Fat* | *Fiber^b^* | *A^c^* | *B1* | *B2* | *B3* | *C* | *Calcium** | *Iron* | *Sodium** |
| **Age group** |  |  |  |  |  |  |  |  |  |  |  |  |
| Toddlers (1-2.9y)  - Boys  - Girls | 100  100 | 0.87  0.87 | ND | 14*  14* | 210 (600)  210 (600) | 0.4  0.4 | 0.4  0.4 | 5 (10)  5 (10) | 13 (400)  13 (400) | 500 (2500)  500 (2500) | 3 (40)  3 (40) | 1 (1.5)  1 (1.5) |
| Children (3-8.9y)  - Boys  - Girls | 100  100 | 0.76  0.76 | ND | 14*  14* | 275 (900)  275 (900) | 0.5  0.5 | 0.5  0.5 | 6 (15)  6 (15) | 22 (650)  22 (650) | 800 (2500)  800 (2500) | 4.1 (40)  4.1 (40) | 1.2 (1.9)  1.2 (1.9) |
| Adolescents (9-17.9y)  - Boys  - Girls | 100  100 | 0.75^d^  0.74^d^ | ND | 14*  14* | 538 (2250)^d^  453 (2500)^d^ | 0.85^d^  0.8^d^ | 0.95^d^  0.85^d^ | 10.5 (25)^d^  10 (25)^d^ | 51 (1400)^d^  47.5 (1400)^d^ | 1300 (2500)  1300 (2500) | 6.8 (42.5)^d^  6.8 (42.5)^d^ | 1.5 (2.25)^d^  1.5 (2.25)^d^ |
| Adults (18-49.9y)  - Men  - Women | 100  100 | 0.66  0.66 | ND | 14*  14* | 625 (3000)  500 (3000) | 1  0.9 | 1.1  0.9 | 12 (35)  11 (35) | 75 (2000)  60 (2000) | 1300 (2500)  1300 (2500) | 6 (45)  8.1 (45) | 1.5 (2.3)  1.5 (2.3) |
| Older adults (≥50y)  - Men  - Women | 100  100 | 0.66  0.66 | ND | 14*  14* | 625 (3000)  500 (3000) | 1  0.9 | 1.1  0.9 | 12 (35)  11 (35) | 75 (2000)  60 (2000) | 1300 (2500)  1300 (2500) | 6 (45)  5 (45) | 1.3 (2.5)  1.3 (2.5) |
|  |  |  |  |  |  |  |  |  |  |  |  |  |
| Lactating women | 160 | 1.05 | ND | 14* | 900 (3000) | 1.4 | 1.3 | 17 (35) | 100 (2000) | 1000 (2500) | 6.5 (45) | 1.5 (2.3) |
| Pregnant women | 135 | 0.88^e^ | ND | 14* | 550 (3000) | 1.2 | 1.2 | 18 (35) | 70 (2000) | 1000 (2500) | 22 (45) | 1.5 (2.3) |

* = Adequate Intake (AI) used as DRI instead of EAR.

ND = no data on EAR or AI.

a = values represented in g/kg/day.

b = values represented in g/1,000 kcal.

c = values represented in μg RAE/day (RAE = Retinol Activity Equivalent).

d = computed from 2 recommendations: EAR or AI (and UL) for 9-13 year and EAR or AI (and UL) for 14-18 year for adolescents.

e = The EAR for pregnancy are only for the second half of pregnancy. For the first half of pregnancy, the protein requirements are the same as those of nonpregnant women. Since there was only information on the trimester pregnant women were in, for women in the 2^nd^ semester the EAR for the second half of pregnancy was used.

**Table X3: Acceptable Macronutrient Distribution ranges**

|  | AMDR (as percent of energy)^a^ | | |
| --- | --- | --- | --- |
| Macronutrient | Children | Children | Adults |
|  | 1-3 y | 4-18 y |  |
| Fat | 30-40 | 25-35 | 20-35 |
| *n*-6 polyunsaturated fatty acid^b^ (linoleic acid) | 5-10 | 5-10 | 5-10 |
| *n*-3 polyunsaturated fatty acid^b^ (αlinoleic acid) | 0.6-1.2 | 0.6-1.2 | 0.6-1.2 |
| Carbohydrate | 45-65 | 45-65 | 45-65 |
| Protein | 5-20 | 10-30 | 10-35 |
|  |  |  |  |

^a^AMDR = Acceptable Macronutrient Distribution Range which is the percent of energy intake that associated with reduced risk of chronic disease, yet provides adequate amounts of essential nutrients.

^b^ about 10 percent of the overall can come from longer-chain n-3 or n-6 fatty acids.
